# Navigating AI feedback in translation training: how text type, proficiency, and attitude shape students’ acceptance behaviors

**DOI:** 10.3389/frai.2026.1727544

**Published:** 2026-03-12

**Authors:** Shiyue Chen, Jie Lou

**Affiliations:** School of Humanities and Foreign Languages, Zhejiang Shuren University, Hangzhou, China

**Keywords:** engagement, feedback, large language models (LLMs), post-secondary education, translator training

## Abstract

This study investigates how undergraduate and graduate translation students in post-secondary education engage with and evaluate Large Language Model (LLM)-generated feedback through a mixed-methods approach, analyzing acceptance rates, influencing factors, decision rationales, and perceived limitations. 78 students majoring in translation (55 undergraduates, 23 postgraduates) completed translation tasks spanning six text types and received ChatGPT-3.5-generated feedback. Participants made binary accept/reject decisions with immediate written rationales, followed by semi-structured interviews to explore evaluative criteria and perceived deficiencies. Quantitatively, participants accepted an average of 68.2% of LLM suggestions, demonstrating receptive yet selective engagement, with no student accepting or rejecting all suggestions. Acceptance was most strongly shaped by text type, with technical and news texts receiving the highest approval and literary and tourism texts the lowest. Baseline AI attitude and proficiency further moderated engagement, as optimists accepted more suggestions than skeptics, higher-proficiency students within each academic level demonstrated greater criticality than their lower-proficiency peers, and postgraduates overall exhibited more selective evaluation than undergraduates. Qualitatively, students accepted feedback that corrected objective errors, improved fluency, or resolved uncertainty, but rejected suggestions due to cultural or contextual misunderstandings, preservation of personal style, unconvincing justifications, or risk aversion in high-stakes texts. Rejection often triggered deeper engagement, including self-revision, external verification, and dialogue with the AI. Thematic analysis revealed key deficiencies in LLM feedback, such as cultural blind spots, stylistic flattening, and contextual myopia. These findings highlight that students’ engagement with LLM feedback is shaped by the interplay of task characteristics, individual dispositions, and domain expertise, underscoring the need for translator training programs to develop feedback systems that are contextually aware, stylistically adaptive, and dialogic in post-secondary translation education.

## 1 Introduction

The recent advent of large language models (LLMs), such as OpenAI’s ChatGPT, has rapidly transformed the landscape of language technology and language learning. Powered by deep learning and trained on vast corpora, these generative AI tools can produce human-like text and handle complex language tasks (Fui-Hoon Nah et al., 2023). In fact, LLMs have already demonstrated translation quality comparable to that of dedicated systems such as Google Translate and DeepL (Mohsen, 2024), thereby challenging traditional assumptions about machine translation. Their capacity for individualized feedback and interactive dialogue suggests considerable pedagogical potential (Sharma et al., 2025). Feedback is widely acknowledged as a cornerstone of effective educational practice, exerting a substantial influence on student learning outcomes (Alderman et al., 2012; Gomis et al., 2023). Within the field of translation pedagogy, prompt and detailed feedback is particularly beneficial for learners, yet it places significant demands on instructors’ time and effort. Traditional feedback delivery, in which teachers must compose individualized comments on student work, is both resource-intensive and burdensome (Jacobsen and Weber, 2025). LLMs present a potential solution to this challenge by offering scalable mechanisms for delivering high-quality feedback at reduced cost. A growing body of research has examined ChatGPT’s ability to generate tailored, timely, and comprehensive feedback (Dai et al., 2023; AlGhamdi, 2024; Steiss et al., 2024; Wang et al., 2024; Tam, 2025). Nonetheless, most existing studies have centered on writing contexts, with relatively limited attention paid to its role in translation education (Xu et al., 2025). Moreover, although several studies suggest that ChatGPT-based feedback can improve translation outcomes in relatively straightforward cases (Cao and Zhong, 2023; Cao and Zhou, 2025), systematic understanding remains limited. However, a critical gap exists between technological capability and pedagogical reality: while instructors increasingly adopt AI tools for feedback delivery, we lack a systematic understanding of whether and how translation students critically engage with such feedback in practice. This gap is consequential, as uncritical acceptance of AI suggestions may reinforce errors or cultural misunderstandings, whereas blanket rejection may cause students to miss valuable learning opportunities. Understanding students’ actual decision-making behaviors, when they accept, when they reject, and why, is therefore essential for designing effective AI-integrated translation pedagogy.

Studies of student attitudes toward AI in language learning reveal mixed patterns. Zhang et al. (2025) found Chinese translation students viewed generative AI as enhancing efficiency but expressed concerns about adequacy and cultural competence. Cao and Zhou (2025) reported that students trusted teacher feedback more than ChatGPT’s suggestions. Alotaibi et al. (2025) demonstrated that perceived usefulness and ease of use predicted ChatGPT adoption in English learning. These findings underscore that acceptance depends on multiple factors, including technical competence, perceived benefits, and trust, yet most research examines general attitudes rather than moment-to-moment decision-making during specific tasks. Moreover, these studies predominantly rely on post-hoc self-reports, introducing hindsight bias and limiting understanding of situated evaluation processes.

Furthermore, research indicates that the effectiveness of feedback is determined not only by its linguistic or technical quality but also by how learners engage with it under varying conditions. In writing contexts, for instance, studies comparing AI-generated and human feedback reveal mixed learner responses depending on task demands and individual characteristics. Escalante et al. (2023) found that English-as-a-new-language students valued feedback that was clear, consistent, and specific. Those who preferred AI feedback often emphasized its detailed explanations and explicit rationales, which were particularly beneficial for lower-proficiency learners seeking concrete guidance. More broadly, in educational contexts, transparency and explicit justification have been shown to enhance trust in AI-generated suggestions (Barnes and Hutson, 2024). Yet acceptance is still shaped by students’ baseline attitudes toward technology: optimistic learners are more likely to trust AI, whereas skeptical learners apply stricter evaluative criteria. In translation tasks specifically, engagement is further conditioned by task type. While LLM feedback on technical texts may be readily adopted due to its surface-level accuracy, students tend to be more cautious with literary texts, where cultural nuance and creative interpretation are critical. Varela Salinas and Burbat (2023) argue that machine translation cannot replace the creativity and cultural sensitivity of human translators, a limitation echoed in learner reports of genre-sensitive deficiencies. Similarly, Abdelhalim et al. (2025) found that both novice and advanced EFL translators tended to reject AI-generated renderings of literary passages when these lacked cultural grounding. These studies suggest engagement is a situated process: proficiency level, task type, and baseline attitude jointly condition whether feedback is trusted, critically examined, or integrated into revision strategies.

Despite these insights, existing research exhibits critical limitations. First, most studies examine behavioral intentions or general perceptions rather than observed acceptance behaviors during actual tasks. Second, research in translation education focuses primarily on output quality or overall perceptions, with limited attention to fine-grained evaluative criteria underlying sentence-level decisions. Third, while frameworks such as TAM have been applied to predict AI adoption in writing and language learning, their application to translation-specific tasks, where accuracy, cultural nuance, and stylistic preservation are paramount, remains underexplored. To address these gaps, this study offers three contributions. First, it captures actual acceptance behaviors at the sentence level, requiring students to explicitly accept or reject each AI suggestion with immediate written rationales, reducing hindsight bias and revealing situated reasoning. Second, it examines how acceptance is shaped by the interaction of baseline attitude (optimist vs. skeptic), proficiency level, and text type, extending beyond simple adoption models to account for conditional trust in translation contexts. Third, it employs mixed methods to quantify acceptance rates and qualitatively unpack evaluative criteria, follow-up strategies, and perceived systemic deficiencies informing decision-making and trust calibration. Specifically, the study investigates Chinese translation students’ engagement with and evaluation of LLM-generated feedback through three guiding research questions:

*RQ1*: To what extent do translation students accept LLM-generated feedback, and how do the baseline attitude, text type, and students’ proficiency shape their acceptance behaviors?

*RQ2*: What evaluative criteria guide translation students’ acceptance or rejection of LLM-generated feedback, and what follow-up strategies do they employ after rejection?

*RQ3*: What perceived deficiencies in LLM-generated feedback underpin students’ mistrust, and how do these deficiencies shape their rationale for rejection and the overall calibration of their trust in the AI?

The findings of this study hold significance for multiple stakeholder communities. For translation educators, understanding the conditions under which students appropriately engage with or resist AI feedback can inform the design of AI-integrated curricula and help identify when human oversight remains essential. For curriculum designers and EdTech developers, empirical evidence of students’ acceptance criteria and perceived deficiencies can guide the development of more contextually-aware, culturally-sensitive, and pedagogically sound feedback systems. For translation students themselves, this research illuminates critical engagement strategies that promote autonomous learning while avoiding uncritical over-reliance on AI tools. Finally, for the broader human-centered AI and trust-and-reliance research community, this work contributes behavioral evidence of how domain-specific expertise, task characteristics, and individual dispositions interact to shape human-AI collaboration in educational contexts, an area of growing importance as AI tools become increasingly embedded in learning environments.

## 2 Literature review

### 2.1 Generative AI in translation education

Feedback has long been regarded as a central pedagogical tool in educational contexts and was originally defined as information delivered by teachers, peers, or textual sources in response to learners’ performance (Gomis et al., 2023; Steiss et al., 2024; Xavier et al., 2025). Within translation education, feedback serves a particularly vital function: it not only supports the achievement of learning objectives but also helps students consider their translations from the standpoint of target readers and cultivate evaluative self-awareness (Cao and Zhou, 2025; Su et al., 2025; Xu et al., 2025). A growing body of research has examined different types of feedback in translator training. For example, Cao and Zhou (2025) demonstrated that written corrective feedback can be especially beneficial for learners with lower L2 proficiency, who tend to benefit more from direct interventions. Lin et al. (2021) likewise found that peer feedback not only enhanced translation quality but also strengthened students’ evaluative capacities. Despite these advantages, the provision of translation feedback is highly time-consuming, and the expansion of class sizes has further intensified teachers’ workload (Steiss et al., 2024). Consequently, instructors are often confronted with the challenge of balancing timeliness with quality when responding to students’ work (Xavier et al., 2025). This difficulty has motivated increasing interest in automated methods of feedback delivery that could alleviate teacher workload without compromising pedagogical effectiveness (Xu et al., 2025).

Against this backdrop, the rise of ChatGPT has introduced new possibilities for feedback provision. Unlike earlier machine translation (MT) systems, neural large language models (LLMs) such as ChatGPT can produce more fluent, context-sensitive output (Chen and Lin, 2025). Research in broader educational contexts has begun documenting the potential of AI-generated feedback to enhance learning outcomes. Dai et al. (2023) found that ChatGPT-generated feedback on academic writing improved revision quality when coupled with explicit guidance on how to interpret suggestions. Alghamdi (2024) reported that AI feedback systems could provide personalized, timely responses at scale, addressing a longstanding challenge in large enrollment courses. Steiss et al. (2024) demonstrated that automated feedback reduced instructor workload by approximately 40% while maintaining comparable pedagogical effectiveness to human feedback for mechanical writing errors. However, these studies also noted that AI feedback tended to be less effective for higher-order concerns such as argumentation quality, cultural appropriateness, and creative expression, limitations that may be particularly salient in translation contexts where such dimensions are central rather than peripheral. Scholars have begun to examine how these tools may be integrated into translator training, for instance, Guo et al. (2025) argue that ChatGPT can foster personalized learning opportunities and provide immediate feedback for student translators. At the same time, professional translator training programs are increasingly incorporating exposure to AI-based resources. Traditional tools such as translation memories and terminology databases are now being supplemented by workshops focused on generative AI literacy. Empirical evidence shows that students often appreciate the efficiency and idea-generation capacities of AI tools while remaining aware of their limitations (Wang J. Q. et al., 2025; Wang L. et al., 2025). Similarly, Abdelhalim et al. (2025) found that while novice learners often favored ChatGPT over Google Translate for translation tasks, more advanced translators tended to be more cautious and slower to adopt the new technology. These findings highlight a broader pedagogical imperative: as AI tools become integral to professional practice, translation education must strike a balance between technical engagement with AI and the cultivation of human judgment. Although there is a growing body of work on AI-assisted feedback in translation learning, systematic investigations into ChatGPT’s role in translation-specific feedback remain limited. Cao and Zhou (2025), for example, compared revisions produced with ChatGPT-generated feedback against those informed by teacher feedback and found differences in the quality of the revised drafts. However, their study primarily focused on translation outcomes and offered little insight into learners’ perceptions of the feedback process or the strategies they employed during revision. As highlighted in broader educational research, the effectiveness of feedback depends not only on its linguistic accuracy or technical quality but also on how learners interpret, engage with, and act upon it (Li, 2014; Sato and Loewen, 2018). To fully understand the pedagogical value of ChatGPT-generated feedback in translator training, it is therefore crucial to examine how students interact with this feedback during the revision process and how they perceive its strengths and limitations.

### 2.2 Student engagement with AI feedback

Understanding how students engage with AI-generated feedback requires attention to their underlying attitudes and beliefs about these technologies. Technology acceptance frameworks, particularly the Technology Acceptance Model (TAM), have proven influential in explaining technology adoption in educational contexts (Dianati et al., 2022; Li et al., 2024; Tam, 2025). Technology acceptance frameworks (TAM) emphasize that perceptions of usefulness, ease of use, and trust determine adoption of new tools (Taherdoost, 2018). Recent studies of ChatGPT in higher education broadly confirm this pattern. For instance, Choudhury and Shamszare (2023) demonstrated that students’ willingness to use ChatGPT increased when they trusted the system’s outputs. Maheshwari (2024) showed that Vietnamese students’ intention to use ChatGPT was strongly driven by perceived usefulness, with trust moderating this relationship. Ma et al. (2025) uncovered that ease of use and usefulness predicted ChatGPT adoption, with trust again exerting a significant moderating effect. Notably, these relationships were stronger among individuals with higher educational backgrounds, suggesting that education shapes how perceptions translate into adoption behavior.

However, human-centered AI research distinguishes between trust as a psychological attitude and reliance as observable behavior. Vereschak et al.’s (2021) methodological review demonstrates that trust, defined as a user’s willingness to depend on an AI system, does not automatically translate into behavioral reliance. Students may report high trust yet exhibit cautious behavior during actual tasks, or conversely, may rely heavily on AI despite low trust due to time pressure or self-doubt. This trust-reliance gap highlights a methodological limitation in existing ChatGPT adoption research, which predominantly measures self-reported trust rather than capturing actual reliance behaviors during task performance.

Baseline attitudes also play an important role: learners who are generally receptive to technology engage more readily with AI feedback (Kashive et al., 2020). In translator training specifically, disciplinary expertise further conditions such attitudes. Wang J. Q. et al. (2025) and Wang L. et al. (2025) found that Hong Kong translation majors valued ChatGPT for its performance gains but approached it critically by drawing on their technical knowledge, whereas non-translation majors relied more heavily on institutional cues and social endorsement when deciding whether to adopt the tool. These studies provide insights into students’ orientations toward AI, yet predominantly rely on self-reported behavioral intentions rather than capturing actual acceptance behaviors during authentic tasks, raising questions about whether stated attitudes predict real-world engagement.

Beyond general attitudes, research in writing and language learning contexts has identified multiple factors that condition how learners engage with AI-generated feedback. Findings from the broader human–AI trust literature also illuminate these patterns. Research consistently shows that novices and experts process AI feedback differently. Novices often exhibit automation bias, readily accepting AI outputs even when flawed, while experts are quicker to identify errors and recalibrate their trust. Banovic et al. (2023) demonstrated that users lacking domain expertise are particularly vulnerable to accepting AI suggestions uncritically, especially when AI expresses high confidence in its outputs. Inkpen et al. (2023) further elaborated on how user expertise moderates human-AI collaboration. Their research demonstrated that domain experts were better able to identify AI errors and selectively integrate suggestions, whereas novices exhibited either excessive deference to AI or blanket rejection following a single error. Importantly, Inkpen et al. (2023) also found that task complexity moderated these patterns, novices were particularly prone to uncritical acceptance in complex tasks where self-evaluation was difficult. By analogy, translation students’ level of expertise likely influences their engagement with AI feedback: advanced students (e.g., MA versus BA level) are more inclined to scrutinize suggestions critically. Abdelhalim et al. (2025) found that Saudi EFL student translators appreciated ChatGPT’s fluency, but advanced students were more sensitive than novices to its mishandling of idiomatic and cultural nuances.

Research in English-as-a-second-language (ESL) writing contexts further illuminates how learners evaluate and engage with automated feedback. Wilson and Czik (2016) found that ESL students particularly valued automated feedback systems that provided detailed metalinguistic explanations alongside error corrections, as these scaffolds helped them understand not just what was wrong, but why. Similarly, Ranalli (2018) demonstrated that explicit, rule-based justifications in automated writing evaluation (AWE) systems enhanced learner trust and uptake, particularly among lower-proficiency students who benefited from concrete guidance. More recently, Yan and Zhang (2024) showed that transparency in AI feedback mechanisms functioned as a key moderator of acceptance in academic writing contexts, though the study also cautioned that overly technical explanations could overwhelm users lacking computational literacy.

Translation text type further shapes how students interact with AI feedback. While LLMs perform well on factual and structurally explicit genres, they continue to struggle with culturally embedded or creative texts. Chen and Lin (2025), for instance, compared ChatGPT, Google Translate, and DeepL on Chinese tourism brochures and found that ChatGPT achieved the highest levels of fidelity and fluency when appropriately prompted, though it still produced occasional semantic distortions in culture-specific passages, underscoring the need for human oversight. In literary translation, LLMs often succeed at grammatical and structural accuracy but fail to capture tone and subtext (Abdelhalim et al., 2025). Karpinska and Iyyer (2023) reported that ChatGPT-3.5 generated translations with fewer grammatical and cohesion errors, yet “critical errors persist,” particularly omissions and mistranslations that undermine the author’s voice. These genre-based limitations align with Salimzadeh et al.’s (2023) findings: LLMs may struggle precisely where students need the most support, creating conditions where uncritical acceptance could be particularly problematic.

The literature demonstrates that students’ engagement with AI feedback is shaped by interactions among trust, reliance behaviors, task characteristics, and expertise. Human-centered AI research establishes that trust and reliance are distinct constructs (Vereschak et al., 2021), with user expertise as a critical moderator: novices are prone to uncritical acceptance when AI projects confidence (Banovic et al., 2023), while experts demonstrate more calibrated engagement following error exposure (Inkpen et al., 2023). Task complexity further conditions these patterns, with heavier reliance on difficult tasks where LLMs may be least reliable (Salimzadeh et al., 2023). Pedagogical interventions that scaffold critical evaluation can foster informed selective adoption (Prabhudesai et al., 2025). In translation contexts, TAM variables influence initial adoption, while text genre modulates situated engagement.

Despite these insights, three critical limitations exist. First, a methodological gap: much research relies on hypothetical scenarios or self-reported surveys rather than measures of actual acceptance during authentic tasks. This introduces hindsight bias and limits understanding of moment-to-moment evaluation processes. Studies documenting general attitudes (Zhang et al., 2025; Alotaibi et al., 2025) or preferences (Cao and Zhou, 2025) provide baseline data but do not capture situated reasoning and real-time decision-making. Second, a contextual gap: while research examines AI feedback in writing (Dai et al., 2023; Escalante et al., 2023) and language learning (Alotaibi et al., 2025), limited attention addresses translation-specific criteria. Translation tasks demand simultaneous attention to linguistic accuracy, cultural appropriateness, and stylistic preservation, dimensions beyond surface-level correctness in writing feedback research. Existing translation studies focus on output quality (Cao and Zhou, 2025) or general perceptions (Abdelhalim et al., 2025) but have not systematically investigated evaluative criteria students apply or follow-up strategies after rejection. Third, a process gap: research has not sufficiently examined how perceived deficiencies recalibrate trust. While studies document advanced learners becoming cautious after encountering errors (Abdelhalim et al., 2025), fine-grained understanding is lacking of which deficiencies (cultural blind spots, stylistic flattening, inadequate explanations) most undermine trust, and how students reconcile overall attitudes with instance-level judgments. Research has not distinguished micro-level acceptance criteria from macro-level trust calibration.

## 3 Methodology

### 3.1 Research design and participants

The present study adopted an explanatory sequential mixed-methods research design, in which quantitative behavioral data were collected and analyzed first to identify patterns of students’ acceptance of AI-generated translation feedback, followed by qualitative data to explain the underlying mechanisms driving these patterns. This design was selected to align explicitly with the three research questions, which required both (a) systematic measurement of acceptance behaviors and (b) in-depth interpretation of learners’ reasoning, perceptions, and decision-making processes. In the quantitative phase, students’ accept/reject decisions, baseline AI attitudes, and interaction logs were analyzed to examine overall acceptance trends and group differences. In the subsequent qualitative phase, written rationales and semi-structured interviews were used to interpret how students evaluated AI feedback in relation to translation quality, task demands, and self-perceived competence. This study attempts to uncover how and why translation students, defined as students formally enrolled in undergraduate or postgraduate programs in Translation or Translation and Interpreting, engage with AI-generated feedback in pedagogical contexts.

The current study was conducted in two comprehensive universities located in China. Both institutions offer undergraduate and postgraduate translation and interpreting programs, situated within Translation Studies, a field encompassing translation theory, practice, pedagogy, and quality evaluation. The B.A. and M.A. programs provide a diverse curriculum including courses such as Theory of Translation, Principles of Translation, Non-literary Translation, Literary Translation, and Computer-Assisted Translation (CAT), equipping students with essential skills for professional practice. Additionally, regular LLM-related workshops and seminars enhanced students’ ability to integrate LLMs into translation learning. Prior to the study, students primarily received delayed, selective human feedback, whereas the AI system provided immediate, sentence-level feedback with consistent explanations, which may have influenced students’ expectations and responses. Participants were recruited via purposive sampling to ensure representation across proficiency levels and program stages, with inclusion criteria: (1) enrollment in undergraduate (years 2–4) or postgraduate (MA years 1–2) Translation Studies programs; (2) at least 1 year of formal translation training; (3) no prior formal exposure to AI-generated translation feedback (see Table 1).

**Table 1:** Demographic information of questionnaire participants (*N* = 78).

Variable	Category	*N*	Percentage
Grade	Undergraduate	55	70.5%
	Postgraduate	23	29.5%
LLM use frequency	Daily	26	33.3%
	Weekly	32	41.0%
	Monthly or less	20	25.6%
Gender	Female	52	66.7%
	Male	26	33.3%

**Figure 1:**
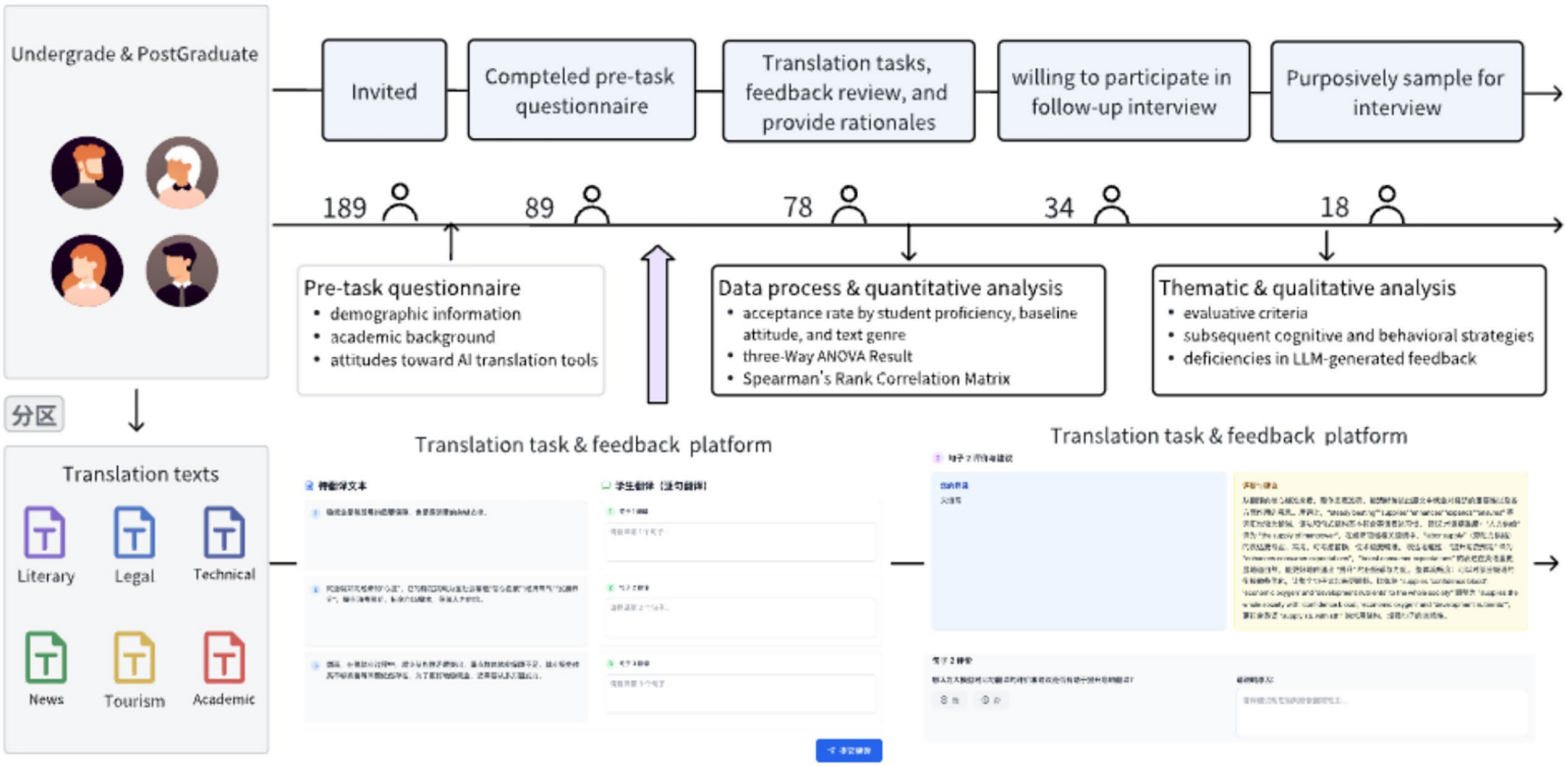
Schematic diagram of the research design.

**Figure 2:**
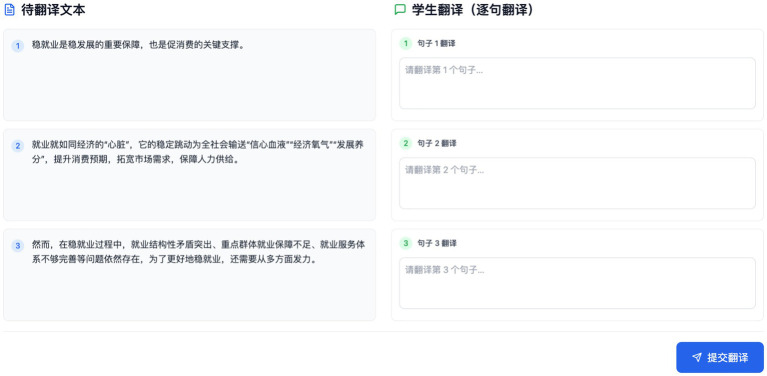
Sample of student translation task.

**Figure 3:**
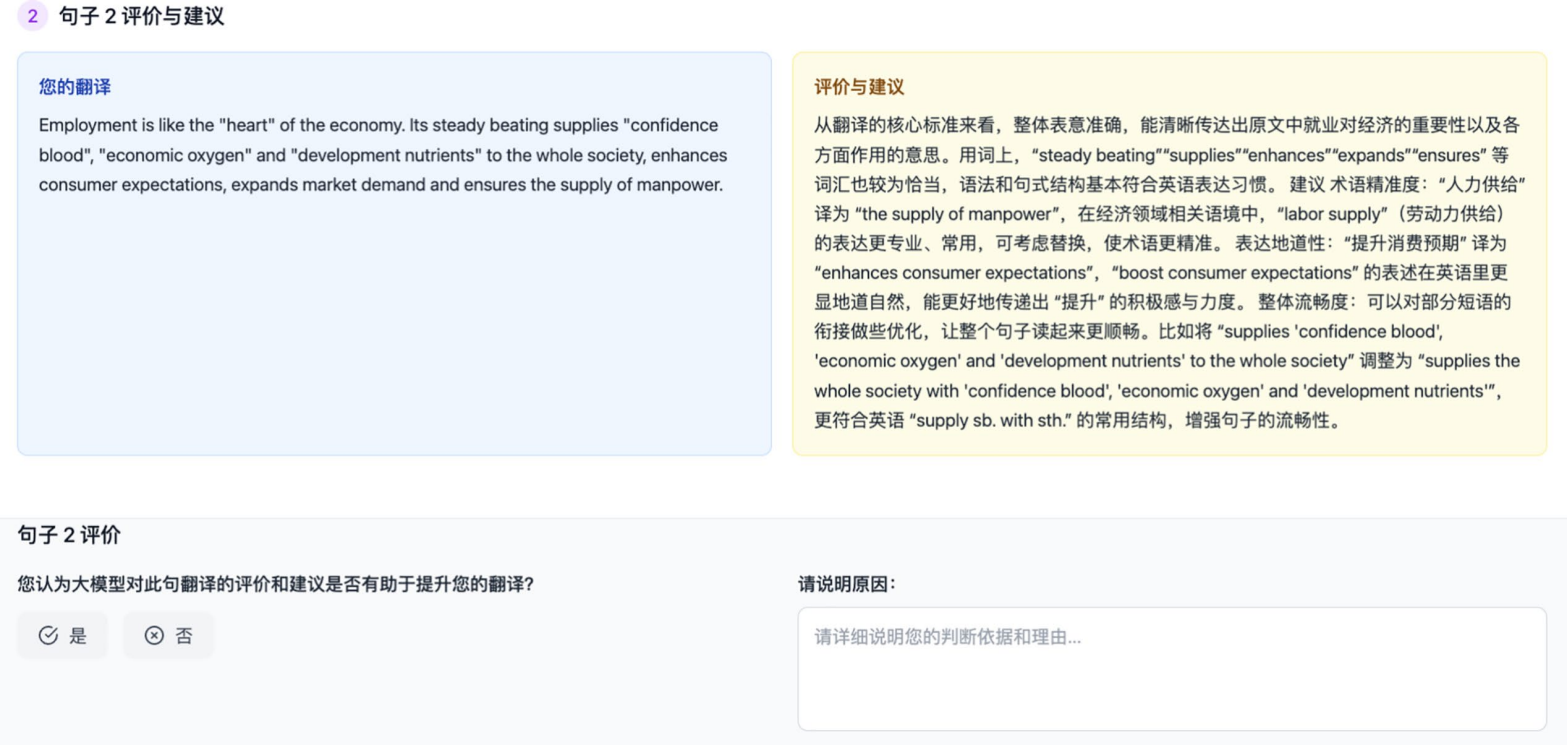
AI-powered translation feedback platform.

Figure 1 illustrates the overall workflow. Initially, *N* = 189 translation students were recruited from the two universities. Participants completed a pre-task questionnaire capturing demographic information, academic background, and baseline attitudes toward AI translation tools. Written informed consent was obtained, and participants were reminded of their right to withdraw. 89 students completed the pre-task questionnaire, and after applying inclusion criteria, 78 participants proceeded to the translation task, supported by an AI-powered translation feedback platform integrated with ChatGPT-3.5 (via OpenAI API, May 2025) (Figures 2, 3). The platform was customized through prompt engineering to generate context-sensitive feedback addressing accuracy, fluency, lexical choice, and coherence, and to provide explanatory rationales for formative purposes. Students could receive feedback, review suggestions, and compare drafts with AI-augmented versions. All data, including interactions, were anonymized and securely stored. Participants were explicitly assured that grades or academic standing would not be affected by participation. The study protocol was approved by the university research ethics board. Prior to the main study, a pilot study with 10 volunteer students (excluded from the final sample) refined task clarity, interface usability, and evaluation questions, confirming task feasibility and comprehension.

The demographics of the 78 survey respondents were 66.7% female (*n* = 52), 33.3% male (*n* = 26), 70.5% undergraduate, 29.5% postgraduate. LLM use frequency was 23.1% daily, 41.0% weekly, 25.6% monthly or less, and 10.3% never. 18 of 34 volunteers were selected for follow-up interviews via purposive sampling (Tables 2, 3). All participants had at least intermediate proficiency in English (as the translation tasks were from Chinese into English) and prior exposure to machine translation (MT) or computer-assisted translation (CAT) tools.

**Table 2:** Students’ proficiency by academic level and course grade.

Academic level	Course grade	Participants
U	A	18
U	B+	22
U	B	15
P	A	8
P	B+	9
P	B	6

Proficiency criteria were based on the “Translation Theory and Practice” course (core for translation majors) to ensure consistency in evaluating translation ability.

**Table 3:** Demographic information of interview participants (*n* = 18).

Pseudonym	Gender	Academic level	LLM use frequency	Grade
P1	Female	U	Weekly	A
P2	Male	P	Daily	B
P3	Female	U	Monthly	B+
P4	Female	U	Weekly	B
…	…	…	…	…
…	…	…	…	…
…	…	…	…	…
…	…	…	…	…
P18	Female	P	Daily	A

U, undergraduate; P, postgraduate.

Baseline attitudes toward LLM were measured using a short Technology Acceptance Model (TAM) scale adapted for AI tools, including perceived usefulness, ease of use, and trust (Mustofa et al., 2025). All TAM items were rated on a 5-point Likert scale (1 = Strongly disagree, 5 = Strongly agree). Participants were qualitatively profiled (e.g., optimist: TAM ≥ 3.5, skeptic: TAM < 3.5) based on TAM scores and comments, which later informed the interpretation of acceptance patterns.

To enhance methodological transparency and clarify the alignment between research questions, data sources, and analytical procedures, Table 4 summarizes the correspondence among research questions, data types, instruments, analytical methods, and expected outputs.

**Table 4:** Alignment of research questions, data, instruments, and analyses.

RQ	Data type	Instrument	Analytical method	Evidence
RQ1	Quantitative behavioral data	AI-powered feedback platform (accept/reject logs), pre-task questionnaire	Descriptive statistics; group comparisons	Acceptance rates; distribution across attitude and proficiency groups
RQ2	Quantitative + qualitative	TAM scale; course grade records; written decision rationales	Comparative analysis; qualitative content coding	Relationships between attitudes, proficiency, and acceptance decisions
RQ3	Qualitative interpretive data	Mandatory written rationales; semi-structured interviews	Thematic analysis; cross-case comparison	Explanatory mechanisms underlying acceptance, rejection, and critical engagement

### 3.2 Task procedures and data collection

The experiment was conducted online via TenCent Meeting on 8 May 2025. Participants were first tasked with translating six short texts (120–200 words each, see Appendix 3) from Chinese into English within 100 min (Table 5). These texts were selected to represent diverse genres, ranging from factual and standardized styles to highly creative and culturally nuanced content, to capture variation in translation challenges and the potential differential impact of AI feedback across contexts of differing complexity, formality, and stylistic demands. To facilitate participants’ work, each text was segmented into sentences for translation, and students translated them sentence by sentence (see Figure 2). Correspondingly, ChatGPT provided its feedback and suggestions on a sentence-by-sentence basis (see Figure 3).

**Table 5:** Text types for translation tasks.

No.	Category	Text characteristics
T1	News report	Clear structure, information-oriented, formal language
T2	Tourism brochure	Culturally rich content, persuasive and vivid style
T3	Technical text	Terminology-heavy, logical, low-context
T4	Academic abstract	Highly standardized, complex sentence structures
T5	Legal clause	Precise wording, rigorous sentence structure, and strong logical coherence
T6	Literary excerpt	Creative expressions, stylistically distinctive

**Figure 4:**
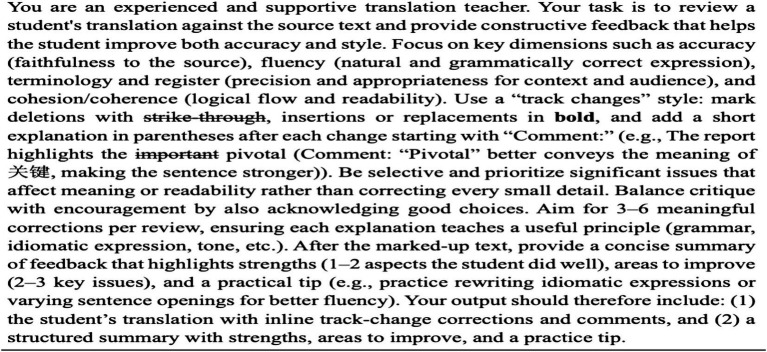
The built-in prompt used to generate ChatGPT feedback.

Two professional translation teachers confirmed text difficulty. Students were instructed to translate independently (dictionaries or online term research allowed), screen sharing ensured transparency, and uncertainties were recorded. After translation, AI feedback was delivered with a consistent prompt (Figure 4) and marked-up suggestions, simulating best-practice pedagogical feedback. We deliberately asked the AI to be somewhat selective and to ensure explanations were given, to simulate what might be considered best practices in feedback. Each student received the AI feedback on their six texts in this manner. It should be acknowledged that while ChatGPT can produce multiple feedback outputs from a single prompt, this study analyzed only the initial response.

Students then entered the feedback reception phase, illustrated in Figure 2. In this interface, the blue section (“Your Translation”) displayed the student’s own English sentence, while the yellow section (“Evaluation and Suggestions”) presented ChatGPT’s proposed revisions, such as suggestions for improving fluency, grammar, or stylistic appropriateness. To the right, a “Sentence Evaluation” panel was provided, where students were asked to make a judgment on the prompt “您认为大模型对此句翻译的评价和建议是否有助于提升您的翻译? (literal translation: Do you think the large language model’s evaluation and suggestions for this sentence help improve your translation?”). They recorded their choice (Yes or No) and then completed the adjacent “Please Explain Your Reason” field, giving a brief justification. This structured interface required students not only to decide whether to accept or reject each suggestion, but also to articulate their reasoning immediately after reviewing the feedback. Such a design reduces hindsight bias and allows us to capture students’ authentic, real-time decision-making processes. In cases of uncertainty, students were permitted to consult external resources, as a professional translator would, and they were encouraged to record such actions in the “Please Explain Your Reason” or mention them during interviews. Additionally, they had the option to open a separate chat window if they wished to seek clarification from the AI, ensuring that the original feedback record remained unaffected. After all texts were completed with decisions logged, 18 students participated in a semi-structured interview (30–45 min). The interview served to delve deeper into their experiences, using their own rationale as a springboard for questions. An interview protocol was developed to ensure systematic coverage of all research questions (RQs) while allowing for in-depth exploration of individual participant experiences (see Appendix 2). A set of pre-formulated questions aligned with the three RQs served as the foundational structure for all interviews. The interviews were conducted primarily in the students’ native language (Chinese) to allow them to express nuances freely, though many students used English for specific translation examples. All interviews were audio-recorded and transcribed for analysis. This two-stage combination of rationale plus interview provided both granular decision-level data and holistic reflections, giving our analysis rich texture.

### 3.3 Data analysis

A mixed-methods analytical framework integrated quantitative and qualitative approaches. The primary quantitative outcome was Acceptance Rate (AR), computed globally (Global AR), by student proficiency (AR-SP), and by text type (AR-TT) using standard formulas:


$$\text{Global}\;\mathrm{AR}=\frac{\sum_{i=1}^{78}\sum_{j=1}^{6}X_{\mathrm{ij}}\;}{78\times 6}\times 100\%$$

where $X_{\mathrm{ij}}$ represents the binary judgment (1 = accepted, 0 = rejected) made by participant i for text type j .

To capture proficiency-related variation, subgroup-specific AR values (AR by Student Proficiency, AR-SP) were calculated for each of the six proficiency-based cohorts (Undergraduate A, Undergraduate B+, Undergraduate B, Postgraduate A, Postgraduate B+, Postgraduate B). For example, the AR for the Undergraduate A group was computed as:


$$\mathrm{AR}-{\mathrm{SP}}_{\text{UndergradA}}=\frac{\sum_{i=1}^{18}\sum_{j=1}^{6}X_{\mathrm{ij}}}{18\times 6}\times 100\%$$

Analogous calculations were performed for the remaining groups. In addition, genre-specific acceptance rates (AR by Text Type, AR-TT) were calculated for each text category using the following formula:


$$\mathrm{AR}-{\mathrm{TT}}_{\text{Text}1}=\frac{\sum_{i=1}^{78}X_{i1}}{78}\times 100\%$$

Three-way ANOVA examined effects of student proficiency and text type, with η2 as effect size and *p* < 0.05. Analyses were conducted in Python (NumPy v2.0.2, Pandas v2.2.3, Seaborn v0.13.2).

The qualitative strand followed Braun and Clarke's (2022) reflexive thematic analysis framework. Two primary sources informed the analysis: students’ written justifications in the feedback decision logs and transcripts from the follow-up interviews. MAXQDA 2022 was used to organize and code the data systematically. The analysis unfolded through iterative phases designed to ensure rigor and depth. Specifically, the six stages of reflexive thematic analysis were applied. The process began with immersion, as the research team repeatedly read the rationales and transcripts, noting initial impressions and collating all “reasons for decision” into a consolidated reference file. This was followed by systematic initial coding, in which discrete segments of text representing a single idea or rationale were assigned descriptive codes. Coding proceeded through both deductive strategies, guided by the research questions and relevant theoretical concepts, and inductive strategies, allowing unanticipated patterns to emerge. While the decision logs typically contained concise explanations, the interviews provided more elaborate accounts that contextualized students’ reasoning. Subsequent phases involved clustering related codes into broader categories and then refining these into themes that directly addressed the research questions. For RQ2, which examined the evaluative criteria underlying students’ judgments, distinct thematic patterns were identified. For RQ3, which focused on perceived shortcomings of LLM feedback, critical comments were grouped into thematic categories. These candidate themes underwent iterative refinement and validation through constant comparison with the raw data to ensure internal coherence and clear differentiation. Reliability was enhanced by having two researchers independently code a subset of the material and resolve discrepancies through discussion. Credibility was further supported through member checking: preliminary thematic summaries were shared with selected participants, who confirmed that the interpretations resonated with their experiences (Shenton, 2004). Final themes were clearly defined and supported with illustrative excerpts from both rationales and interviews in the results section.

## 4 Findings

### 4.1 RQ1

The overall analysis revealed that participants accepted an average of 68.2% (SD = 8.5%) of the LLM’s suggestions (see Table 6). This finding indicates that while most suggestions were perceived as useful, participants did not adopt them indiscriminately. Notably, no student accepted all the AI’s recommendations; even those who expressed relatively high levels of trust rejected at least a few suggestions. Conversely, even the most skeptical participants incorporated some of the AI’s input.

**Table 6:** Acceptance rate by student proficiency and genre.

Baseline attitude	AR (%)	SD
Optimists	74.6	6.9
Skeptics	59.9	7.5
Student proficiency		
UA	52.3	7.1
UB+	69.4	6.8
UB	78.1	5.9
PA	45.7	8.3
PB+	65.2	7.5
PB	72.9	6.2
Genre		
T1	79.5	5.8
T2	50.8	9.1
T3	82.1	6.4
T4	75.8	7.0
T5	54.2	8.8
T6	48.6	9.5
Overall	68.2	8.5

Building on this general trend, we next examined the role of students’ initial attitudes toward AI. To this end, we incorporated a baseline attitude measure from the pre-task TAM questionnaire, classifying participants as either optimists or skeptics. The comparison revealed a clear attitudinal effect: optimists demonstrated higher acceptance rates (*M* = 74.6%, SD = 6.9%) compared to skeptics (*M* = 59.9%, SD = 7.5%). This 14.7-percentage-point gap underscores that pre-interaction attitudes toward generative AI were a strong predictor of feedback uptake.

In addition to baseline attitudes, students’ proficiency also exerted a significant influence. Participant proficiency was assessed based on academic level and performance in the core course Translation Theory and Practice, which evaluates accuracy, fluency, terminology use, and problem-solving in translation tasks. While no standardized test was administered, the course grade served as a valid program-specific indicator of translation competence. Future research may incorporate standardized assessments to capture finer distinctions across proficiency levels. Results revealed a consistent pattern within each academic level: students with higher course grades (A) demonstrated greater selectivity than those with lower grades (B+, B). Among undergraduates, grade A students showed the lowest acceptance rate (52.3%, SD = 7.1%), followed by B + (69.4%, SD = 6.8%) and B (78.1%, SD = 5.9%). Similarly, among postgraduates, grade A students were the most selective (45.7%, SD = 8.3%), followed by B + (65.2%, SD = 7.5%) and B (72.9%, SD = 6.2%). This within-level pattern indicates that higher proficiency (as measured by course grade) corresponded to more critical evaluation of AI feedback. Importantly, however, an additional pattern emerged when comparing across academic levels: postgraduates as a whole exhibited lower acceptance rates than undergraduates, even when controlling for course grade. For instance, postgraduate B students (72.9%) were more selective than undergraduate B students (78.1%), and postgraduate A students (45.7%) were notably more critical than undergraduate A students (52.3%). This cross-level difference suggests that academic level—reflecting greater exposure to translation training, theoretical frameworks, and disciplinary socialization—functions as an independent moderator of AI feedback engagement beyond course-specific performance. In other words, proficiency operates through a dual mechanism: (1) within-level effects, where higher course grades predict greater criticality, and (2) cross-level effects, where advanced academic standing (MA vs. BA) fosters more cautious evaluation regardless of grade.

Complementing these individual-level influences, genre-specific analysis further highlighted the contextual sensitivity of feedback acceptance. Among the six text types, technical (T3) and news texts (T1) achieved the highest ARs, at 82.1 and 79.5%, respectively, followed by academic texts (T4) (75.8%). In contrast, literary (T6) and tourism texts (T2) exhibited the lowest uptake, with ARs of 48.6 and 50.8%, respectively. Legal texts (T5) occupied an intermediate position (54.2%). Taken together, these genre effects demonstrate that students were more receptive to LLM-generated feedback when working with fact-oriented or structurally explicit content, whereas culturally embedded or stylistically nuanced texts elicited greater skepticism. This interpretation aligns with prior research (Liu et al., 2025), which has shown that learners perceive creative and culture-loaded genres as less amenable to automation, thereby exercising greater caution when integrating LLM-generated feedback.

**Figure 5:**
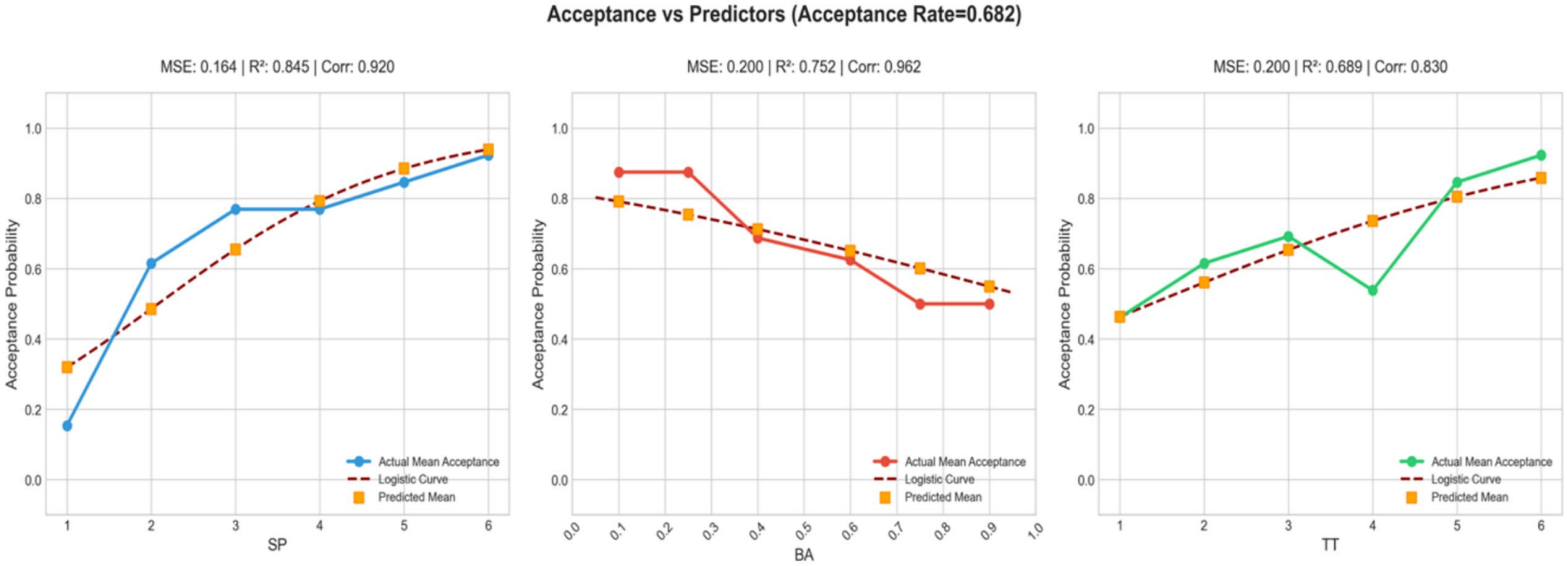
Model-predicted vs. observed acceptance probabilities.

To assess the relative contributions of the three determinants, student proficiency (SP), baseline attitude (BA), and text type (TT), we conducted logistic regression analyses, with acceptance probability of LLM-generated feedback as the dependent variable and the three predictors as independent variables. Figure 5 displays the predicted acceptance probabilities generated by the model alongside the observed data. The close alignment between observed and predicted values suggests that the model captures the acceptance patterns effectively. Across all three predictors, the coefficient of determination (*R*^2^ > 0.75) indicates that the regression models explain more than 75% of the variance in acceptance probability, while the high correlation coefficients (Corr > 0.8) further confirm the strong predictive validity of the fitted models. In practical terms, this means that learner proficiency, prior attitude toward AI, and the nature of the translation task each exert systematic and measurable effects on whether students accept or reject LLM-generated feedback. The subsequent three-way ANOVA (Table 7) corroborates these findings by showing statistically significant main effects for SP, BA, and TT.

**Table 7:** Three-way ANOVA results.

Effect	df	*F*	*p*	η^2^	95% CI for η^2^	Observed power
SP	2, 144	12.8	<0.001	0.043	[0.025, 0.061]	0.95
TT	5, 360	28.5	<0.001	0.112	[0.089, 0.135]	>0.99
BA	1, 144	22.4	<0.001	0.061	[0.041, 0.081]	0.98

Within this framework, all three factors exert meaningful influence, though their relative magnitudes differ. TT demonstrated the strongest effect on acceptance behavior (*F*(5, 360) = 28.5, *p* < 0.001, *η*^2^ = 0.112), indicating a medium-to-large effect size. This underscores the critical role of genre characteristics, suggesting that structural and cultural properties of texts shape students’ willingness to integrate AI-generated suggestions to a considerable degree. BA also emerged as a robust predictor (*F*(1, 144) = 22.4, *p* < 0.001, *η*^2^ = 0.061), corresponding to a medium effect size. This finding confirms that students’ pre-existing dispositions toward generative AI substantially modulate their acceptance decisions. Finally, SP showed a statistically significant but comparatively smaller effect (*F*(2,144) = 12.8, *p* < 0.001, *η*^2^ = 0.043), classified as a small-to-medium effect size. Although weaker than text type or baseline attitude, this result suggests that linguistic expertise remains an important determinant: students with advanced proficiency adopt a more selective and critical stance toward AI feedback, whereas those with lower proficiency are more likely to rely on AI assistance.

**Figure 6:**
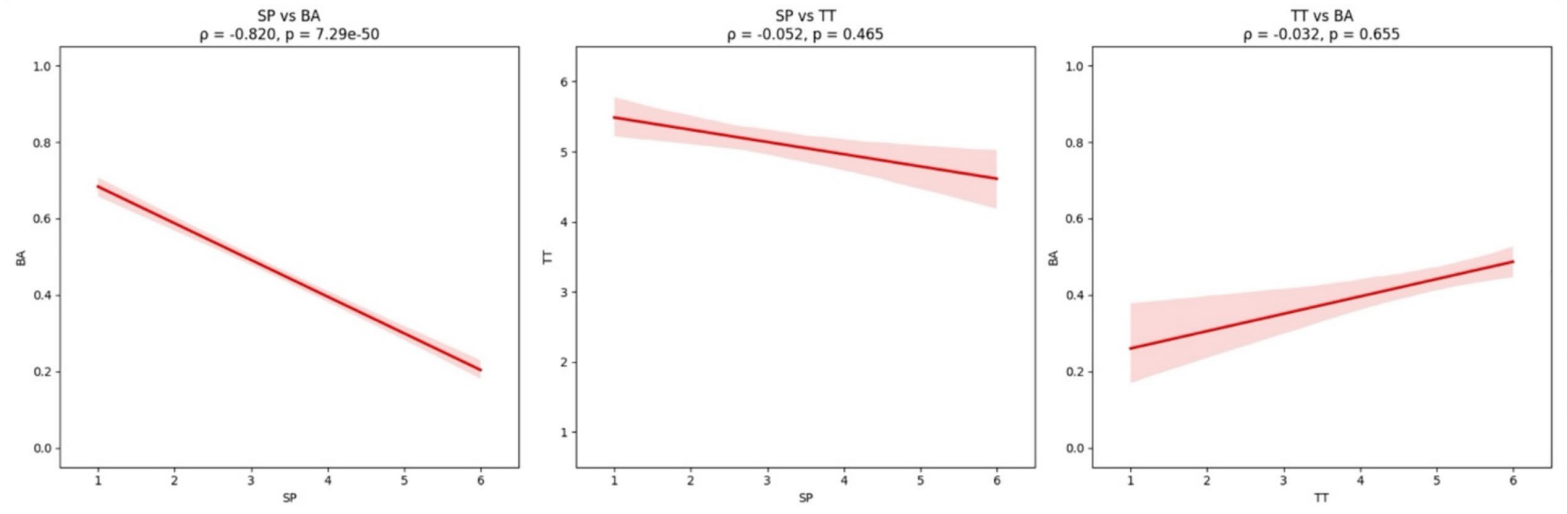
Correlation scatter plot.

To further clarify interrelationships among these factors, Spearman’s rank-order correlations were computed (see Table 8). Results revealed a strong negative correlation between SP and BA, *ρ* = −0.82, *p* < 0.001, indicating that higher-proficiency students were substantially more likely to hold skeptical attitudes toward generative AI feedback. In contrast, no significant correlation was observed between SP and TT, *ρ* = −0.05, *p* = 0.465, nor between TT and BA, *ρ* = −0.03, *p* = 0.655. This negative relationship between SP and BA was further visualized in Figure 6, where the scatterplots and fitted regression lines illustrate the robust inverse association between proficiency and baseline attitude, in contrast to the flat, non-significant trends for SP × TT and TT × BA.

**Table 8:** Spearman’s rank correlation matrix.

Variables	SP (p)	TT (p)	BA (p)
SP	1.000 (0.000)	−0.052 (0.465)	−0.820 (**7.29e-50**)
TT	−0.052 (0.465)	1.000 (0.000)	−0.032 (0.655)
BA	−0.820 (**7.29e-50**)	−0.032 (0.655)	1.000 (0.000)

Bold p-values indicate statistical significance at the *p* < 0.001 threshold. The p-value of 7.29e-50 corresponds to the Spearman correlation between Student Proficiency (SP) and Baseline Attitude (BA) (*ρ* = −0.820), reflecting an exceptionally strong level of significance that far exceeds conventional thresholds. All other pairwise correlations (SP×TT: *ρ* = −0.052, *p* = 0.465; TT×BA: *ρ* = −0.032, *p* = 0.655) were non-significant. SP = student proficiency; TT = text type; BA = baseline attitude.

**Figure 7:**
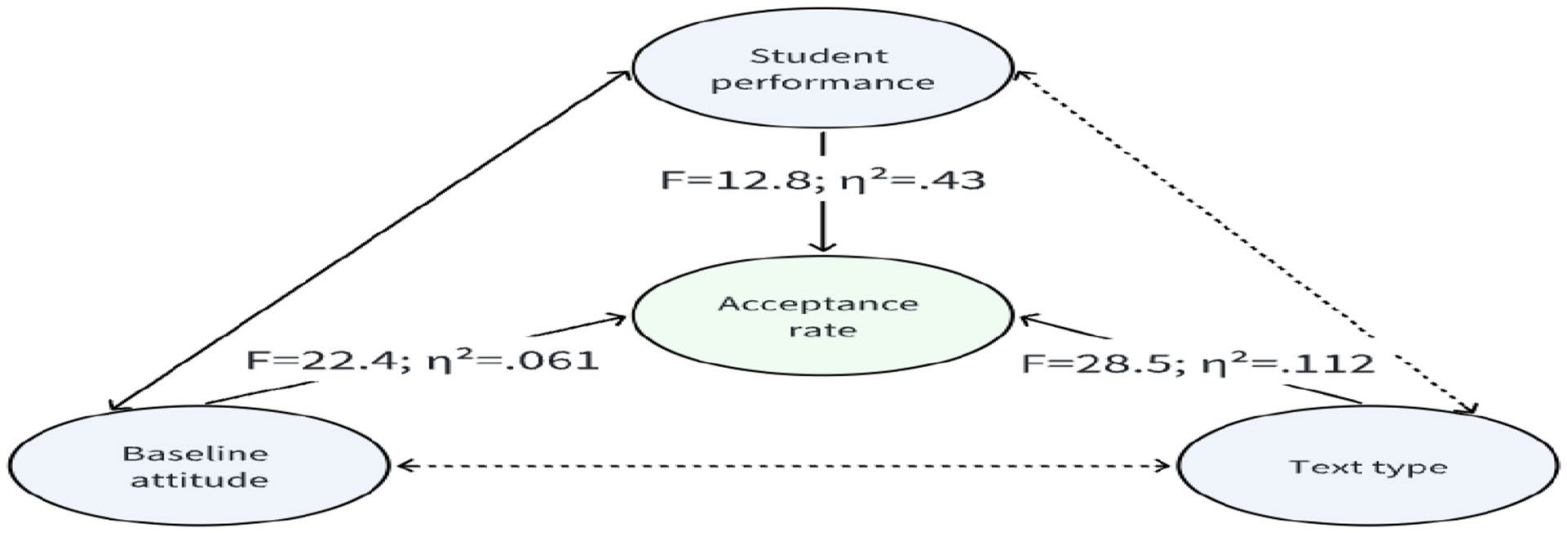
Diagram of factors influencing the acceptance rate of LLM-generated feedback.

Synthesizing across these analyses, Figure 7 illustrates that students’ acceptance of LLM-generated feedback was shaped independently by three key determinants: text type (TT), baseline attitudes (BA), and proficiency (SP). Among these, text type exerted the strongest influence, followed by baseline attitudes, while proficiency had the comparatively weakest effect. Notably, a negative correlation emerged between proficiency and baseline attitudes, suggesting that greater linguistic expertise was associated with more cautious or skeptical orientations toward generative AI. The results, therefore, demonstrate that the uptake of AI feedback is not reducible to a single dimension but reflects the distinct and combined effects of textual characteristics, learners’ dispositions, and linguistic competence, highlighting the multifactorial and context-dependent nature of human–AI interaction in translation learning.

### 4.2 RQ2

**Figure 8:**
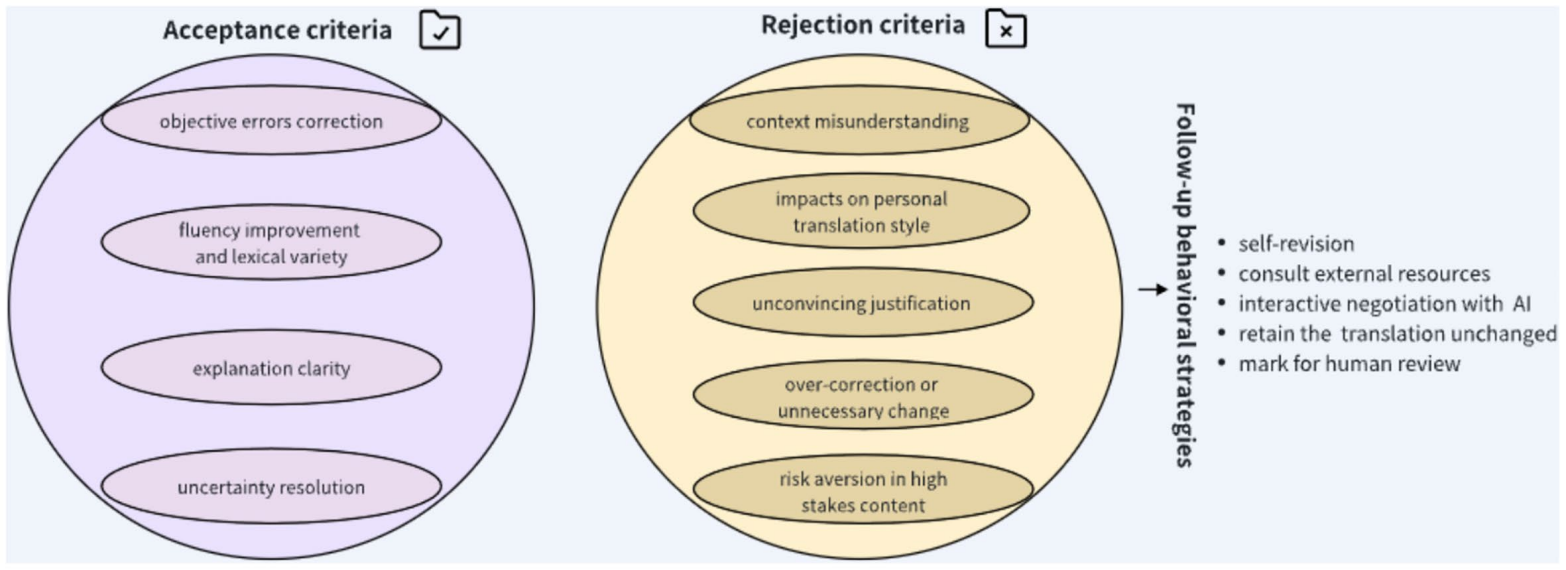
Core criteria for students’ judgment of LLM-generated feedback.

Analysis of students’ written justifications and interview responses revealed several key criteria guiding their acceptance of AI-proposed changes (see Figure 8). These criteria encompass correction of objective errors, improvement in fluency and lexical choice, clarity of explanation, and resolution of uncertainty. The most frequently cited reason for acceptance was the correction of clear, objective errors. When the AI identified mistakes such as grammatical inaccuracies, spelling errors, mistranslations of factual details (e.g., dates or proper nouns), or obvious lexical misuse, students almost invariably adopted the suggested revision. For instance, if a student omitted the plural “-s” and the AI corrected it, acceptance was immediate. Similarly, in cases where idioms were misinterpreted, participants acknowledged the AI’s correction as “clearly the correct meaning.” One participant explicitly stated, “I translated the idiom incorrectly; GPT’s suggestion is clearly the correct meaning.” Interviews suggest that students viewed the AI as a reliable safeguard against mechanical errors, particularly in grammar and spelling, which they assumed the AI was highly competent at handling. As one student commented, “GPT is like having a super spell-checker and grammar guru looking over my shoulder.” These objective corrections were particularly valued in fact-oriented genres such as technical and news texts, consistent with RQ1 results, where these genres had the highest acceptance rates. Conversely, such errors were less common in literary and tourism texts, which partly explains why students in those genres were more cautious and selective. Notably, such errors were also less common among higher-performing students (A-level), whose lower acceptance rates reflected fewer clear-cut errors and more stylistic suggestions.

Another major acceptance criterion was the perceived enhancement of fluency, naturalness, and lexical variety. Students frequently embraced suggestions that made their translations sound more idiomatic, stylistically polished, or concise, especially when they were uncertain about their original phrasing. For example, a student translated a Chinese phrase literally as “enter the eyes of the public,” and GPT suggested “capture public attention.” The student accepted this change, noting, “GPT’s phrase is more idiomatic English.*”* In other cases, verbose sentences were streamlined by the AI; students typically accepted these changes when meaning was preserved while clarity improved. One participant commented, “It’s like having a native speaker refine my sentences.” Synonym replacements were also common, such as changing “very big change” to *“significant change,”* and were generally accepted if students understood and agreed with the alternative. However, baseline attitude influenced this criterion: optimists were more willing to trust lexical refinements, even when suggestions were stylistically minor, while skeptics often rejected them as unnecessary overcorrections.

The inclusion of a clear explanation significantly increased acceptance likelihood. When the AI provided a rationale such as “This sentence is in passive voice, which makes it a bit clunky; using active voice here makes it clearer who is doing the action.” Students were more inclined to trust and adopt the suggestion. One participant remarked, “When GPT explained its thinking, I felt more confident adopting the suggestion—it was like a mini-lesson.” Explanations were valued not only for justifying individual changes but also for offering transferable insights, thereby reinforcing learning. This aligns with findings in explainable AI research that transparency fosters trust (Barnes and Hutson, 2024; Shafik, 2024). Conversely, suggestions lacking clear justification were more frequently rejected. Thus, clarity of explanation emerged as a key criterion: revisions accompanied by persuasive reasoning, particularly those referencing grammar norms, usage conventions, or fidelity to source meaning, were most often accepted. Notably, students with higher proficiency levels were especially attentive to explanation quality; they sometimes rejected changes if the reasoning was vague or inaccurate, reflecting their stronger metalinguistic awareness compared to lower-performing peers (Spellerberg, 2016).

Finally, acceptance often stemmed from the AI’s ability to resolve students’ uncertainty about their original translation. When participants were unsure, particularly with idioms, culturally embedded expressions, or domain-specific terms, the AI’s confident suggestion functioned as a default solution. Rationale included remarks such as, “Accepted—I’m not confident my translation is correct, and GPT’s suggestion seems reasonable.” This happened in segments where the student wasn’t sure of the best translation (perhaps they had marked it as uncertain during initial translation). Similarly, one interviewee admitted, “For the second text, I honestly didn’t know how to translate that idiom. My attempt was a shot in the dark. So, when I saw GPT’s version, I thought: ‘It probably knows better than me,’ and I accepted it.” Some students double-checked these suggestions via quick online searches, which reinforced their trust. This pattern reflects a reliance on AI as an authoritative source in areas where students lack confidence, though it raises concerns about potential over-reliance. This shows that students use AI feedback to reduce uncertainty (Chang et al., 2025; Nazaretsky et al., 2022), if they were on the fence, a confident suggestion from the AI gives them an “anchor” to latch onto. Here again, baseline attitude shaped responses: optimists were more likely to accept the AI’s anchor without hesitation, while skeptics tended to seek external confirmation or rely on their own judgment. Genre also played a role, with uncertainty-driven acceptance particularly prominent in literary and tourism texts, where ambiguity and cultural nuance made students feel less secure in their initial choices.

Students also declined a substantial proportion of the AI’s suggestions. The most prevalent reason was the perceived inability of the AI to capture context or meaning accurately. When students believed a suggestion distorted the source text or failed to convey its intended nuance, they tended to reject it. This issue was particularly evident in culturally embedded or contextually rich passages. For instance, in the tourism text, a line included a metaphor tied to local tradition. Several students rendered it literally but poetically, whereas GPT proposed a more generic phrasing. Students rejected GPT’s version, arguing that “GPT doesn’t understand the cultural significance here”. One student wrote, “Rejected—GPT’s change loses the underlying cultural reference; it’s not just about accuracy, it’s about the feeling/tone which GPT missed.” Another participant emphasized, “This was about a historical reference—I doubt GPT really knew the background, whereas I remembered it from class, so I kept my translation. I didn’t trust GPT on that.” Similar concerns arose in literary text translations where GPT idiomatic or creative headlines with literal ones. Such genre-driven skepticism was particularly pronounced among higher-performing students and baseline skeptics, who consistently highlighted AI’s limitations in cultural sensitivity and stylistic competence (Wang J. Q. et al., 2025; Wang L. et al., 2025).

A second theme concerned the preservation of personal translation style or intent. Many students viewed translation as an expressive act and resisted AI suggestions that altered their deliberate stylistic choices. GPT often converted flowery or nuanced sentences into simpler, more standard phrasing. Students who had intentionally crafted their wording rejected such changes. One rationale read, “Rejected—My wording is unconventional but deliberate to match the tone of the source. AI’s version is bland.” In interviews, one participant described rejecting a correction because it removed alliteration: “I know I added a tiny flourish not in the original, but it felt right to convey the mood. GPT doesn’t do flourish—it made it too plain.” This pattern was especially visible among advanced students with stronger grades, who emphasized their stylistic agency and confidence (Reeve, 2013). By contrast, lower-performing students were more inclined to accept AI’s version, perceiving it as the safer or more authoritative option.

Lack of trust or unconvincing justification was another frequent reason for rejection. When AI suggestions lacked explanations or offered vague rationales, students were reluctant to adopt them. For example, GPT might propose a synonym accompanied only by “improved clarity,” which students found insufficient. As one interviewee remarked, “If it doesn’t tell me why, I suspect it might be a preference rather than a necessity.” Some explanations even undermined trust by being inaccurate or irrelevant. One participant recalled an instance where GPT claimed a phrase was “ambiguous,” though the student knew it was not: “That made me lose confidence that GPT knows what it’s talking about.” Afterward, the student scrutinized subsequent suggestions more critically. These findings indicate that explanations must be both clear and correct; a flawed explanation can erode trust more than none at all (Zhang et al., 2025). Interestingly, skeptics were quicker to downgrade trust after a single flawed explanation, whereas optimists tended to give the AI a “second chance.”

Another common reason was the perception of over-correction or unnecessary change. Students objected to revisions that seemed to “fix what isn’t broken.” Many comments reflected frustration with stylistic tweaks that offered negligible improvement: “Rejected—difference is negligible; my original is fine,” or “Rejected—GPT is just rephrasing for the sake of it; I don’t see an improvement.” For instance, GPT changed “according to statistics” to “according to the statistics,” or “can be seen as” to “could be seen as.” Students judged such edits as trivial and retained their originals. This pattern suggests students adopted a principle common in professional editing, avoid changes unless they add substantive value, while also resisting cognitive overload from unnecessary modifications (Zhang et al., 2025). As one participant explained, *“I thought, why am I changing this? It was perfectly acceptable before.* Here again, proficiency differences mattered: stronger students were more likely to dismiss trivial edits, while weaker students sometimes accepted them, interpreting AI’s authority as sufficient justification.

Finally, risk aversion in high-stakes content contributed to rejection, particularly in legal or technical passages. When an AI suggestion seemed risky, such as a bold rephrasing in a contract clause, students preferred to err on the side of caution. One student explained, “For legal terms, I’d rather be safe with what I know, even if GPT could be right. If I’m wrong, I can explain I stuck to the source; if I used GPT’s and it’s wrong, that’s harder to justify.” Comments like “Rejected, not comfortable using this phrasing in a legal sentence, I don’t fully understand it” illustrate this conservative approach. Students prioritized fidelity and defensibility over fluency in sensitive contexts, echoing professional norms in translation where liability risks shape decision-making (Emery, 2004). This caution was particularly strong among skeptics and high-performing students, who weighed potential risks more heavily than optimists or lower-performing peers.

The current research also examined students’ behaviors after rejecting AI suggestions. Analysis revealed several distinct follow-up strategies. Self-Revision emerged as a frequent response: students often rejected the AI’s change but still reevaluated their original, sometimes producing a third alternative. For example, GPT might suggest changing A → B; the student rejects B but, upon reflection, modifies A into C. Students acknowledged this in the interview: “GPT’s suggestion wasn’t good, but it made me think about that sentence again, and I decided to tweak it differently.” From a pedagogical standpoint, this indicates that even unadopted AI feedback can indirectly stimulate learning and text improvement (Guo et al., 2025). A second behavior involved consulting external resources. When rejections stemmed from uncertainty, students often verified their choice by checking dictionaries, bilingual corpora, or class notes. One student, unsure about a legal term, consulted a legal glossary after rejecting GPT’s alternative. Another validated usage via a bilingual corpus, “I didn’t trust GPT on this, so I double-checked elsewhere.” Rationale annotations confirmed such actions: “(after rejecting, I looked this up in Oxford English Dictionary, my version is indeed acceptable).” These behaviors suggest a combination of skepticism and research-oriented decision-making. Some students engaged in interactive negotiation with the AI by querying GPT for clarification. For example, when uncertain, a student asked, “Why did you suggest changing X to Y? The original term X is a specific concept, is Y accurate here?” In several cases, GPT defended its choice or conceded the error, occasionally leading students to reverse their rejection. As one participant reflected, “I treated it like asking a teacher, ‘are you sure this is better?’ and depending on the answer I decided.” Such interactions illustrate a shift from one-way feedback consumption to two-way engagement with AI feedback (Yaseen et al., 2025).

In other cases, students opted to retain their original translation unchanged, particularly when rejections were based on stylistic preference or redundancy. These students exhibited strong confidence in their own linguistic competence and typically did not pursue further revision. Some noted their rationale, “I think it’s fine,” without elaboration. This behavior was most common among advanced students. Finally, a small subset of students marked certain cases for subsequent human review. For example, a student rejecting GPT’s rendering of an idiom wrote, “Rejected, I will check with Prof. X about this idiom.” Although infrequent, this behavior underscores that students continue to value human expertise as the ultimate arbiter in ambiguous cases, positioning AI feedback as a supplementary rather than authoritative resource. In sum, rejection did not necessarily mark the termination of engagement with feedback. For many students, it initiated deeper cognitive and research processes, including self-revision, external verification, and interactive dialogue with the AI. Others asserted confidence in their own solutions or deferred to human expertise. These patterns highlight that rejection is not merely a negative outcome but often an active and constructive form of engagement with AI feedback.

### 4.3 RQ3

Through thematic analysis of students’ logs and interview comments, several recurring deficiencies emerged in their evaluation of LLM-generated feedback. These deficiencies were often cited as reasons for rejecting suggestions or as general critiques during reflective interviews. Cultural blind spots were among the most frequently mentioned issues. Students observed that the LLM lacked cultural awareness and contextual sensitivity, which led to feedback that was inappropriate for culturally rich content. For example, in Text 2 (tourism brochure), one line referenced a local myth; many students felt GPT’s suggestion “simplified the myth to a bland statement,” thereby stripping away cultural nuance. Another remarked during the interview, “It tried to correct a line about a historical figure’s nickname, but it clearly didn’t understand the story behind it.” Such comments underscore an inherent limitation: LLMs operate on linguistic patterns rather than cultural comprehension. Consequently, when a translation task required sensitivity to historical or cultural connotations, students perceived the AI’s feedback as unreliable. As one interviewee succinctly summarized, “GPT has knowledge, but no cultural sense.”

Another prominent theme was stylistic flattening. Students frequently criticized GPT for homogenizing their translations, describing its preferred style as “standard,” “safe,” or “stiff.” While such changes often improved grammatical accuracy and clarity, they also diminished the stylistic richness of the original student work. For instance, one student noted, “All of GPT’s suggestions made my translation sound the same as everything else, it took away my unique wording.” Similarly, another remarked, “It’s like it has one voice, a textbook voice. That’s not always suitable.” These observations resonate with prior findings in MT research that neural systems tend toward high-frequency, normalized patterns, thereby reducing variation and nuance (Zhang et al., 2022; Ul Qumar et al., 2025). As one interviewee warned, “GPT’s corrections were grammatically correct, but devoid of personality—if we all used GPT’s suggestions, all our translations would read similarly.” This reflects a broader pedagogical concern: students feared that overreliance on LLM feedback might homogenize learning outcomes and stifle creativity (Kumar et al., 2025).

Students also reported contextual limitations, noting that GPT’s feedback sometimes failed to consider broader discourse or earlier decisions within the same text. Despite being provided with paragraph-level input, the model occasionally produced suggestions that clashed with previous choices. One student explained, “GPT suggested using ‘they’ in a sentence, but in the previous sentence I had already clarified the subject, so repeating it was unnecessary, GPT seemed to forget what was just said.” Another pointed to terminological inconsistency: “The suggestion is not wrong in isolation, but I need consistency, GPT isn’t consistent with what I used in other parts.” This tendency to optimize locally rather than globally creates coherence problems, such as tone shifts or contradictory terminology. As one interviewee reflected, “I treated some suggestions skeptically because I felt GPT isn’t reading my whole translation like a human would, it’s looking at bits and might mess up consistency.” This stands in contrast to the decision-level focus in RQ2: here, students articulate a systemic deficiency, criticizing GPT’s lack of holistic reading ability, which they perceived as a fundamental distinction between human and AI reviewers.

A further limitation concerned mechanical and uninformative explanations. Although GPT was instructed to justify its suggestions, students often found these explanations formulaic and superficial. Many defaulted to generic phrases such as “this sounds more natural” or “this is clearer.” One student expressed frustration: “Every suggestion was justified by ‘more natural’ that doesn’t tell me much. Why is it more natural? In what way? That explanation is kind of robotic.” Another added, “It felt like copy-paste rationale, not really engaging with my text.” More concerning, some explanations were inaccurate, such as misidentifying a grammar rule, which further eroded trust. Students indicated that meaningful, context-specific rationales would make feedback more pedagogically valuable. In RQ2, the lack of convincing justification often led to rejection of a suggestion; in RQ3, however, students critiqued the explanatory style of the LLM itself as “mechanical” and insufficient for learning, thereby questioning its role as a tutor rather than a corrector.

Finally, students highlighted rigidity and lack of dialogic flexibility in the feedback process. GPT often presented its suggestions in an authoritative, one-size-fits-all manner, e.g., “Replace X with Y” without acknowledging alternatives or uncertainty. Several students contrasted this with human feedback, which tends to offer multiple options or frame suggestions as possibilities rather than mandates. One participant observed, “GPT didn’t suggest multiple options; a teacher might say, you could say it like this or like that. It just gave one solution, so if I didn’t like it, that was it.” This rigidity limited opportunities for collaborative decision-making and positioned the AI as an inflexible authority rather than a dialogic partner. Whereas RQ2 captured how students negotiated with individual suggestions (accepting or rejecting them), RQ3 findings reveal students’ broader critique of the interactional mode of AI feedback. This highlights that their concerns extend beyond translation accuracy to the very dynamics of human-AI collaboration in educational settings.

## 5 Discussion

This study reveals that translation students engaged with LLM feedback through a process of critical collaboration, accepting approximately two-thirds of suggestions while rejecting the remainder based on evaluative criteria rooted in translation competence and genre sensitivity. This pattern of selective engagement diverges from both extremes reported in prior literature: near-universal acceptance in automated writing feedback studies (Hibert, 2019; Thi et al., 2023; Shin and Lee, 2025) and categorical skepticism in some language learning contexts (Kushmar et al., 2022). The theoretical significance of this finding lies in what it reveals about how disciplinary contexts shape cognitive orientations toward AI tools. Whereas writing students may approach automated feedback primarily as error-correction mechanisms, translation students, trained in principles of equivalence, fidelity, and cultural mediation, activate more complex evaluative schemata that foreground interpretive judgment alongside linguistic accuracy. This disciplinary difference suggests that AI reliance is not merely a function of general technology acceptance (as TAM would predict) but is fundamentally mediated by domain-specific epistemic frameworks that define what constitutes authoritative knowledge in a given field.

The three-factor model (text type, baseline attitude, proficiency) revealed differential effects, with text type exerting the strongest influence, followed by baseline attitude, and proficiency demonstrating the comparatively weakest effect. This hierarchy challenges conventional assumptions in educational technology research that learner characteristics (proficiency, motivation) typically dominate task characteristics in shaping technology use (Granić and Marangunić, 2019). The primacy of text type in our findings instead foregrounds the epistemological nature of translation problems: technical texts involve primarily retrieval and application of terminological knowledge, domains where LLMs demonstrably excel, whereas literary texts demand creative synthesis and cultural interpretation, precisely the areas where current architectures struggle (Karpinska and Iyyer, 2023; Abdelhalim et al., 2025). This aligns with Salimzadeh et al.’s (2023) framework on task complexity in human-AI decision-making, which demonstrates that users rely more heavily on AI in cognitively demanding tasks where independent evaluation is difficult. However, our findings reveal a critical paradox: translation students turned to AI feedback most readily for text types (technical, news) where they needed it least, while exercising caution precisely where AI assistance might have been most valuable (literary, tourism texts with cultural nuance). This paradox underscores a fundamental mismatch between LLM competence profiles and learner support needs, suggesting that current AI systems are optimized for tasks where human expertise is already robust rather than domains requiring genuine cognitive scaffolding.

The strong predictive role of baseline attitude (η^2^ = 0.061, medium-to-large effect) provides empirical support for TAM frameworks (yet our findings critically refine how attitude functions in actual task performance). Rather than operating as a rigid determinant, attitude functioned as a cognitive filter with conditional thresholds: even skeptical students accepted unambiguous grammatical corrections, while optimistic students rejected suggestions when cultural or stylistic concerns arose. This conditional nature of attitude effects suggests that TAM models, which typically predict behavioral intentions through linear pathways (perceived usefulness → attitude → intention → behavior), may oversimplify the cognitive dynamics of situated technology use. Our evidence indicates that attitudes establish default stances (optimists begin with openness, skeptics with scrutiny), but instance-level decisions are ultimately governed by task-specific evaluative criteria (e.g., whether a suggestion addresses an objective error vs. stylistic preference). This finding extends Cheah and Li’s (2020) observation that acceptance is a dynamic, case-by-case process, revealing the specific mechanism through which attitudes modulate but do not determine engagement: they set acceptance thresholds rather than dictate outcomes.

The comparatively weaker effect of proficiency (η^2^ = 0.043, medium effect) requires careful interpretation, as the underlying pattern is more nuanced than a simple linear relationship. Our analysis revealed that proficiency operated through a dual mechanism: within-level effects (where higher course grades predicted greater selectivity within both undergraduate and postgraduate groups) and cross-level effects (where postgraduates demonstrated more critical evaluation than undergraduates, even when controlling for course grade). This dual structure helps explain the statistical pattern: while proficiency showed consistent directionality, higher expertise corresponded to lower acceptance; the effect size was moderated by the interaction between academic level and course performance. Theoretically, this finding extends Inkpen et al.’s (2023) observation that domain experts better identify AI errors and selectively integrate suggestions. However, our data refine this insight by disaggregating two dimensions of expertise: (1) task-specific translation competence (captured by course grades), which enables recognition of linguistic and cultural errors, and (2) disciplinary maturity (captured by academic level), which reflects internalization of professional norms, theoretical frameworks, and metacognitive strategies. The within-level consistency (A students most selective in both groups) demonstrates that translation competence directly shapes critical evaluation. This suggests that disciplinary maturity effects are strongest among higher-performing students who can effectively mobilize advanced theoretical knowledge, while among lower-performing students, the gap between academic levels narrows, possibly because both groups rely more heavily on AI assistance when facing uncertainty. The educational context further amplifies this pattern: assessment pressures and disciplinary socialization in graduate programs incentivize demonstration of autonomy and critical reflection, distinguishing academic contexts from professional settings where efficiency may dominate (Škobo and Petričević, 2023). The strong negative correlation between proficiency and baseline attitude (*ρ* = −0.82, *p* < 0.001) further suggests that expertise cultivation in translation education may inherently foster skepticism toward AI, as students internalize disciplinary norms emphasizing human judgment in interpretive tasks. Importantly, however, this correlation likely captures the combined effect of both course-level competence and program-level maturity, as higher-performing postgraduates would be most strongly represented in the “high proficiency, skeptical attitude” quadrant.

A central theoretical contribution of this study is the concept of productive friction, which reframes rejection of AI feedback not as a failure of uptake but as a pedagogically generative process. This concept operates analogously to “desirable difficulties” in cognitive science (Bjork and Bjork, 2020): just as retrieval challenges and spaced repetition enhance long-term retention by forcing deeper encoding, friction with AI feedback appears to trigger elaborative processing. Evidence of this generative process emerged clearly in our data: rejections frequently prompted self-revision (students modified their own translations after disagreeing with AI), external verification (dictionary consultation, corpus checks), interactive negotiation (querying the AI for clarification), or deferral to human expertise (marking segments for teacher review). These behaviors signal active knowledge construction rather than passive consumption of feedback. Critically, these finding challenges the prevailing optimization discourse in educational AI research, which often frames success in terms of maximizing acceptance rates or minimizing editing time. By foregrounding productive friction as a learning mechanism, we propose a paradigm shift: the goal of AI integration in translation education should not be frictionless automation but rather pedagogically orchestrated collaboration, where students develop meta-competence to strategically engage with, critically evaluate, and selectively override AI suggestions. The theoretical mechanism underlying productive friction can be understood through the lens of self-regulated learning theory (Zimmerman, 2002). When students reject AI feedback, they are compelled to engage in forethought (analyzing why the suggestion is inadequate), performance monitoring (comparing AI output against their own translation knowledge), and self-reflection (evaluating the rationale for their decision). This metacognitive cycle transforms disagreement from a simple binary choice into a recursive process of knowledge refinement. For instance, our interview data revealed that even when students ultimately retained their original translations, the act of justifying rejection deepened their understanding of translation principles. One participant noted, “Having to explain why I didn’t trust GPT’s cultural interpretation made me think harder about what ‘cultural fidelity’ actually means in practice.” This aligns with Prabhudesai et al.’s (2025) findings that students explicitly taught to audit LLM outputs demonstrated significantly greater awareness of AI limitations and more calibrated reliance patterns. However, our study extends this insight by documenting that such critical engagement can emerge organically from task structure (requiring written rationales for decisions) even without explicit instructional scaffolding, suggesting that productive friction is an intrinsic affordance of human-AI interaction when learners possess sufficient domain knowledge to contest outputs.

Acceptance patterns, in contrast, were guided by a principle of “bounded trust,” students extended confidence to AI conditionally, contingent on the objectivity of the feedback and the transparency of its reasoning. Corrections of clear, objective errors (grammatical mistakes, terminological inaccuracies) were embraced almost universally, reflecting recognition that AI excels at pattern-matching tasks within well-defined linguistic rules (Sidhu et al., 2024). This finding validates Banovic et al.’s (2023) observation that users lacking domain expertise are particularly vulnerable to accepting AI suggestions uncritically when AI projects confidence, yet crucially, our data show that even skeptical students accepted objective corrections, suggesting that error clarity overrides dispositional skepticism. The mediating role of explanation quality provides strong pedagogical support for Explainable AI principles (Barnes and Hutson, 2024; Shafik, 2024). Students frequently described substantive explanations as “mini-lessons,” echoing findings that transparent justifications enhance trust. However, our evidence both validates and qualifies existing claims about explainability: while Shafik (2024) suggests that explanations almost uniformly improve trust, our data reveal a more conditional relationship. Poorly constructed or superficial rationales (“this sounds more natural”) undermined confidence, and inaccurate explanations triggered trust recalibration, wherein students scrutinized subsequent suggestions more critically. This finding demonstrates that explanation quality, not merely its presence, is critical for pedagogical uptake, a distinction with significant implications for AI design, suggesting that systems must prioritize substantive, contextualized justifications over generic templates.

Rejection patterns further illuminate the boundaries of appropriate AI reliance. Students articulated clear criteria for declining feedback, most commonly citing misinterpretation of cultural context or nuance, cultural references flattened, poetic phrases reduced to banal expressions. These critiques provide crucial ecological validation for computational research demonstrating LLM fragility in cultural reasoning (Karpinska and Iyyer, 2023) and stylistic adaptability (Wang J. Q. et al., 2025; Wang L. et al., 2025). However, they also reveal learners’ implicit theorization of what constitutes effective pedagogical feedback: not merely accuracy or fluency, but context-sensitivity, preservation of translator voice, and alignment with interpretive intent. This user-centered perspective extends beyond computational metrics (e.g., BLEU scores) to encompass pedagogical affordances such as scaffolding interpretive reasoning and enabling iterative negotiation, design requirements that emerge from situated practice rather than technical benchmarking.

The systemic deficiencies students identified, cultural blind spots, stylistic homogenization, contextual myopia, mechanical explanations, and limited dialogic flexibility, converge on a fundamental constraint of current LLM architectures: they optimize for local linguistic plausibility rather than global coherence or interpretive depth. This limitation is not merely technical but epistemological: LLMs lack the situated, embodied understanding that enables human translators to navigate ambiguity, negotiate cultural difference, and make ethically informed decisions about representation (Wang J. Q. et al., 2025; Wang L. et al., 2025). Consequently, while LLMs can reliably support surface-level accuracy and fluency, their rigidity and limited dialogic scaffolding restrict their role as substitutes for human tutors. This finding reinforces the pedagogical imperative articulated in RQ3: AI tools in translation education must be positioned not as autonomous feedback providers but as collaborative resources whose limitations are explicitly pedagogized, enabling students to develop the meta-competence to recognize when to trust, contest, or transcend AI outputs. Ultimately, this study challenges the prevailing optimization discourse that frames AI success in terms of maximizing acceptance rates. By demonstrating that rejection often triggered deeper cognitive engagement, self-revision, external verification, and meta-cognitive reflection, we propose that the goal of AI integration should not be frictionless automation but pedagogically orchestrated collaboration. Success should be measured not by uptake rates but by indicators of critical engagement, metacognitive growth, and long-term evaluative autonomy. This reframing has profound implications for curriculum design: rather than teaching students to efficiently adopt AI suggestions, translation pedagogy should cultivate environments where contesting AI outputs is recognized as a marker of developing expertise, where disagreement becomes a site of learning, and where students acquire the adaptive competence to navigate the evolving landscape of human-AI collaboration in professional translation practice.

## 6 Implications

For translation pedagogy, our findings call for a shift from treating AI as a productivity aid to positioning it as an object of critical inquiry. Students’ trust in AI-generated feedback is highly conditional and context-dependent, underscoring the need for deliberate instructional support in developing evaluative judgment. Pedagogical interventions should explicitly address both the strengths and limitations of generative models, for example, emphasizing that while LLMs are generally reliable for grammatical correction and surface-level fluency, they frequently struggle with cultural nuance and stylistic intent. Structured activities that require students to justify acceptance or rejection decisions, combined with targeted workshops on prompt formulation and output evaluation, can foster the metacognitive awareness necessary for effective human–AI collaboration.

These findings also clarify the evolving but irreplaceable role of human instructors in AI-integrated translation education. While AI can substantially reduce instructor workload in domains governed by objective rules (e.g., grammar, terminology, fluency), students consistently identified deficiencies in cultural adaptation, stylistic nuance, and contextual interpretation, areas where expert human judgment remains indispensable. Instructors therefore continue to play a critical role in supervising culturally sensitive passages, cultivating individual translator voice, and adjudicating high-stakes texts (Guo and Wang, 2024). Beyond these traditional responsibilities, instructors must now explicitly teach AI literacy, guiding students to evaluate feedback using domain-specific criteria and to recognize common failure modes. Without such scaffolding, lower-proficiency learners may overaccept AI suggestions, whereas advanced learners may develop unwarranted skepticism. These patterns point to a tiered hybrid model: AI-led feedback for routine technical tasks, AI supplemented by peer discussion for general informational texts, and AI combined with authoritative instructor review for culturally nuanced or high-stakes translation tasks.

For the design of AI-enhanced translation tools, our results highlight the importance of transparency, user agency, and contextual sensitivity. Feedback systems should move beyond single corrective outputs to provide explanatory rationales, alternative phrasings, and explicit signals of uncertainty, reflecting the dialogic nature of human tutoring. Genre-sensitive feedback mechanisms, adjusting intervention depth for technical versus literary texts, could further enhance pedagogical effectiveness and learner trust, particularly in addressing cultural blind spots and stylistic consistency. These insights also extend to prompt engineering for educational feedback. Prompts should encourage exploratory, dialogic interaction rather than authoritative correction, inviting students to weigh alternatives, justify decisions, and co-construct understanding with the AI. Such designs transform feedback from a one-way directive into a scaffolded learning dialogue.

At the curricular and policy level, the findings underscore the urgency of systematically integrating generative AI literacy into translator training. Programs should equip students not only to use AI efficiently but also to critically evaluate its outputs, supported by clear ethical guidelines distinguishing low-stakes applications from contexts requiring rigorous human verification. Formal instruction on ethical risk management in AI-assisted translation is essential for preparing students for professional practice. Finally, this study points to several directions for future research. Longitudinal studies are needed to examine how trust, reliance, and evaluative strategies evolve with growing expertise, while cross-linguistic and cross-cultural investigations can test the generalizability of these findings. Together, these avenues highlight a dual imperative: advancing AI capabilities while simultaneously developing pedagogical frameworks that ensure generative AI functions as a constructive partner, rather than a substitute, in translator education.

## 7 Limitations

This study has several limitations that should be acknowledged. First, the absence of independent expert evaluation of AI feedback correctness and final translation quality. While we used course grades as a proficiency indicator, we cannot definitively determine whether students’ rejection decisions were justified by superior evaluative judgment or represented missed opportunities for improvement without expert assessment of both AI suggestion quality and student translation outcomes. Similarly, without expert benchmarking of AI suggestions, we cannot distinguish instances where students appropriately evaluated feedback from cases of uncritical acceptance or unwarranted rejection. Future research should incorporate expert assessment of both AI output correctness and student translation quality to provide objective measures of engagement appropriateness. Second, the study was conducted with Chinese translation students translating from Chinese into English at two universities. The findings may not generalize to other language pairs, educational contexts, or cultural settings where attitudes toward technology and pedagogical norms differ. Third, the experimental design used a single AI model (ChatGPT-3.5) with standardized prompts. Different LLMs or variations in prompt engineering might yield different feedback quality and, consequently, different acceptance patterns. Fourth, the study captured engagement at a single point in time. Longitudinal research is needed to examine how students’ acceptance behaviors and trust calibration evolve with extended exposure to AI feedback and accumulated experience with its strengths and limitations. Fifth, this study operationalized individual differences primarily through baseline attitude (optimist vs. skeptics) and proficiency level. While these factors significantly predicted acceptance behaviors, learners’ psychological profiles are far more complex. Cognitive styles (field-dependent vs. field-independent), epistemic beliefs (absolutist vs. relativistic views of knowledge), self-efficacy in translation, and metacognitive awareness may interact with AI engagement in ways not captured by our binary attitude classification. This complexity underscores the continued need for human teachers who can adapt pedagogical approaches to diverse learner profiles. Future research should incorporate multidimensional psychological assessments to better understand the individual factors shaping human-AI collaboration. Finally, while the mixed-methods design provided rich insights into students’ decision-making processes, the reliance on self-reported rationales introduces potential bias. Students may have rationalized their decisions post-hoc or provided socially desirable explanations rather than revealing authentic reasoning.

## 8 Conclusion

This study offers a comprehensive account of how translation students engage with feedback generated by LLM. Participants demonstrated a pattern of selective rather than unconditional trust in the system. Importantly, acceptance decisions were shaped by a hierarchy of influences: text genre emerged as the most decisive factor, with factual and objective genres eliciting greater reliance, followed by students’ baseline attitudes toward AI, where more optimistic learners were more receptive, and finally by proficiency level, with more experienced students showing heightened criticality. The qualitative analysis further illuminated these dynamics. Students tended to accept corrections that clearly resolved linguistic errors, enhanced fluency, or were accompanied by convincing explanations. Conversely, they rejected suggestions perceived as culturally insensitive, stylistically reductive, inadequately justified, or unnecessarily risky. Notably, rejection did not signify disengagement: in many cases, students pursued additional cognitive strategies such as self-revision or independent research, suggesting that even disregarded feedback can catalyze deeper learning. Taken together, these results highlight the dynamic interplay between AI-generated suggestions, student judgment, and task context. While students valued LLMs as reliable tools for formal accuracy checks, they remained skeptical of their capacity to address creativity and cultural nuance. This has clear pedagogical implications: translation educators should equip students with the critical literacy needed to engage with AI judiciously, while developers of feedback systems should prioritize explainability, contextual sensitivity, and adaptability. Ultimately, the integration of LLMs into translator training will depend on cultivating calibrated trust, enabling learners to discern when to rely on AI support and when to exercise independent expertise, thereby maximizing the benefits of human, AI collaboration without undermining educational or translational quality.

## Data Availability

The raw data supporting the conclusions of this article will be made available by the authors, without undue reservation.
