# Supplementary material for: Navigating AI feedback in translation training: how text type, proficiency, and attitude shape students’ acceptance behaviors
Source: Front Artif Intell. 2026 Mar 12;9:1727544. doi: 10.3389/frai.2026.1727544 (PMC13018157; doi:10.3389/frai.2026.1727544)
Supplement: Supplementary file 1 [file Data_Sheet_1.docx]

**Appendix 1**

**Pre-Task Questionnaire**

**Informed Consent**
Before you begin, please read the following information carefully:

Your participation is completely voluntary. You may choose not to answer any question.

You may withdraw from the study at any time without penalty, and your data will not be used.

All responses will be kept strictly confidential and used for research purposes only.

By proceeding, you indicate that you have read this statement and voluntarily agree to participate in the study.

**Section A: Demographic and Academic Background**

**A1.** Age: ____ years

**A2.** Gender: □ Male □ Female

**A3.** Academic level: □ 2nd-year undergraduate □ 3rd-year undergraduate □ Final-year undergraduate  □ Master’s (Year 1) □ Master’s (Year 2)

**A4.** Years of formal translation training (including university-level courses): ____ years

**A5.** Self-rated English proficiency:

1 – Beginner 2 – Lower-intermediate 3 – Intermediate 4 – Upper-intermediate 5 – Advanced

**A6.** Have you previously used any of the following tools? (Select all that apply)

□ Machine Translation (e.g., Google Translate, DeepL)

□ Large Language Models (e.g., ChatGPT)

□ Computer-Assisted Translation (CAT) tools (e.g., Trados, MemoQ)

□ Terminology management tools

□ Other: ________

**A7.** Frequency of AI-assisted translation use in the past 6 months:

1 – Never 2 – Monthly or less  3 – Weekly 4 – Daily

**Section B: Adapted Technology Acceptance Model (TAM) for AI Translation Tools**

**Instructions**: The following statements refer to AI tools used in translation (e.g., ChatGPT, Gemini). Please indicate your level of agreement on a scale from 1 (Strongly disagree) to 5 (Strongly agree).

**Perceived Usefulness (PU)**

PU1. Using AI translation tools can improve the quality of my translations.

PU2. AI translation tools can help me translate more efficiently.

PU3. AI translation tools can enhance my understanding of complex source texts.

PU4. AI translation tools can provide valuable suggestions that I would not think of myself.

**Perceived Ease of Use (PEOU)**

PEOU1. I find AI translation tools easy to learn.

PEOU2. Interacting with AI translation tools is clear and understandable.

PEOU3. I can use AI translation tools without the need for much technical support.

PEOU4. It is easy for me to incorporate AI translation tools into my translation workflow.

**Trust in AI Tools (TR)**

TR1. I believe AI translation tools can be relied on to provide accurate suggestions most of the time.

TR2. I believe AI translation tools handle cultural and contextual nuances appropriately. TR3. I trust AI translation tools to support me in producing high-quality translations.

**Behavioral Intention to Use (BI)** (Optional TAM extension)

BI1. I intend to use AI translation tools more frequently in the future.

BI2. I would recommend AI translation tools to other translation students.

**Response Scale:** 1 – Strongly disagree 2 – Disagree 3 – Neutral 4 – Agree 5 – Strongly agree

**Appendix 2**

**Semi-Structured Interview Guide**

**Part 0 – Warm-up and Rapport Building**

0.1 Could you briefly describe your overall experience completing the translation tasks with AI feedback? 0.2 Before starting this study, had you ever used AI-based feedback in your translation work? If so, in what ways?

**Part 1 – Decision-Making on LLM-Generated Feedback**

**Core Questions**

1.1 When you reviewed the AI feedback, what general principles or criteria guided your decision to accept or reject a suggestion?

1.2 Can you recall examples of suggestions you accepted immediately? What made them convincing?

1.3 Can you recall examples of suggestions you rejected immediately? What led you to reject them?

1.4 In cases where you were unsure, what did you do next?

**Part 2 – Perceived Deficiencies in AI Feedback**

**Core Questions**

2.1 Were there any recurring issues you noticed in the AI’s suggestions?

2.2 Did you ever feel the AI lacked cultural or contextual understanding? Could you give examples?

2.3 How would you describe the style of the AI’s feedback? Was it helpful, too mechanical, too generic, or something else?

2.4 How did you feel about the explanations the AI provided for its suggestions? Were they clear and detailed enough?

**Part 3 – Overall Reflection and Future Use**

**Core Questions**

3.1 Looking back at the whole process, how would you summaries your trust in AI feedback?

3.2 What do you see as the main strengths of AI feedback for translation students?

3.3 What do you see as its main weaknesses or limitations?

3.4 Under what conditions would you like to use such AI feedback regularly in your translation learning or professional work?

**Part 4 – Closing**

4.1 Is there anything else about your experience with the AI feedback that we haven’t discussed, but you think is important?

**Appendix 3**

*Text 1:* 坚持就业优先政策导向，充分发挥市场和政府作用，合力创造更多就业机会。把就业作为经济社会发展的优先目标，推动财政、货币、产业、就业等政策协同发力，提高发展的就业带动力。结合就业市场变化，打通政策落实梗阻，完善就业服务体系，升级公共就业服务平台，避免出现重“政策出台”，轻“落地效果”，导致政策红利不能充分释放的现象。积极培育灵活就业、新就业形态，完善灵活就业人员社保政策，拓宽就业“缓冲带”，增强其就业抗风险能力。

*Text 2:* 位于满觉陇村、石屋洞前一带。满觉陇俗称满家弄，明清时盛产桂花，为西湖著名赏桂胜地。抗日战争杭州沦陷期间，桂花树被大量砍伐，仅剩少数农家屋前屋后几株老树。抗日战争胜利后稍有恢复。1950年后，新种植一批桂树，至1955年前后，连同老树，数达万株，其中树龄最长的约200年，最大植株盛花年可收桂花一石，桂花成为当地村民重要的经济收入。一代传一代，终于造就了这一片“金粟世界”。

*Text 3:* 插入电池之前，请确保电池和电池盒乾燥且清洁。否则可能会影响电池的接触和防水能力。插入电池后，请确保电池盒盖已牢固扣紧。请注意，盖正确紧固后，红色标记不应可见。确保 USB-C 连接埠盖和镜头保护盖安装牢固并紧固，并且防水密封清洁。否则会影响设备的防水能力。产品上所有孔位均採用内建防水材质。请勿戳孔。

*Text 4:* 随着“大数据、云计算”时代的到来以及传 感器技术的发展，工业系统中能够获取的监测数 据越来越多。传统的浅层 ML 算法很大程度上依 赖于专家先验知识与信号处理技术，难以自动处 理和分析海量的监测数据。而深度学习作为神经网络发展而来的一项新技术，以其强大的特征提取能力为训练海量数据提供了一种解决思路。

*Text 5:* 学位授予单位应当根据学位评定委员会授予学士、硕士、博士学位的决议，公布授予学位的人员名单，颁发学位证书，并向省级学位委员会报送学位授予信息。省级学位委员会将本行政区域的学位授予信息报国务院学位委员会备案。

*Text 6:* 凤姐儿笑道：“这也不难．你把才下来的茄子把皮了，只要净肉，切成碎钉子，用鸡油炸了，再用鸡脯子肉并香菌，新笋，蘑菇，五香腐干，各色干果子，俱切成钉子，用鸡汤煨干，将香油一收，外加糟油一拌，盛在瓷罐子里封严，要吃时拿出来，用炒的鸡瓜一拌就是。
